# Exploration of nutritional and bioactive peptide properties in goat meat from various primal cuts during *in vitro* gastrointestinal digestion and absorption

**DOI:** 10.5713/ab.23.0352

**Published:** 2024-04-01

**Authors:** Pichitpon Luasiri, Papungkorn Sangsawad, Jaksuma Pongsetkul, Pramote Paengkoum, Chatsirin Nakharuthai, Saranya Suwanangul, Sasikan Katemala, Narathip Sujinda, Jukkrapong Pinyo, Jarunan Chainam, Chompoonuch Khongla, Supaluk Sorapukdee

**Affiliations:** 1 School of Animal Technology and Innovation, Institute of Agricultural Technology, Suranaree University of Technology, Nakhon Ratchasima 30000, Thailand; 2 Program in Food Science and Technology, Faculty of Engineering and Agro-industry, Maejo University, Chiang Mai 50290, Thailand; 3 Faculty of Agriculture at Kamphaeng Saen, Kasetsart University, Kamphaeng Saen Campus, Nakhon Pathom 73140, Thailand; 4 Faculty of Agricultural Technology, Valaya Alongkorn Rajabhat University under the Royal Patronage, Pathumthani 13180, Thailand; 5 Department of Applied Biology, Faculty of Sciences and Liberal Arts, Rajamangala University of Technology Isan, Nakhon Ratchasima 30000, Thailand; 6 Office of Administrative Interdisciplinary Program on Agricultural Technology, School of Agricultural Technology, King Mongkut’s Institute of Technology Ladkrabang, Bangkok 10520, Thailand

**Keywords:** Bioactive Peptide, Bioavailability, Gastrointestinal Digestion, Goat Meat, Protein

## Abstract

**Objective:**

This research aims to explore the nutritional and bioactive peptide properties of goat meat taken from various primal cuts, including the breast, shoulder, rib, loin, and leg, to produce these bioactive peptides during *in vitro* gastrointestinal (GI) digestion and absorption.

**Methods:**

The goat meat from various primal cuts was obtained from Boer goats with an average carcass weight of 30±2 kg. The meat was collected within 3 h after slaughter and was stored at −80°C until analysis. A comprehensive assessment encompassed various aspects, including the chemical composition, cooking properties, *in vitro* GI digestion, bioactive characteristics, and the bioavailability of the resulting peptides.

**Results:**

The findings indicate that the loin muscles contain the highest protein and essential amino acid composition. When the meats were cooked at 70°C for 30 min, they exhibited distinct protein compositions and quantities in the sodium dodecyl sulfate-polyacrylamide gel electrophoresis profile, suggesting they served as different protein substrates during GI digestion. Subsequent *in vitro* simulated GI digestion revealed that the cooked shoulder and loin underwent the most significant hydrolysis during the intestinal phase, resulting in the strongest angiotensin-converting enzyme (ACE) and dipeptidyl peptidase-IV (DPP-IV) inhibition. Following *in vitro* GI peptide absorption using a Caco-2 cell monolayer, the GI peptide derived from the cooked loin demonstrated greater bioavailability and a higher degree of ACE and DPP-IV inhibition than the shoulder peptide.

**Conclusion:**

This study highlights the potential of goat meat, particularly cooked loin, as a functional meat source for protein, essential amino acids, and bioactive peptides during GI digestion and absorption. These peptides promise to play a role in preventing and treating metabolic diseases due to their dual inhibitory effects on ACE and DPP-IV.

## INTRODUCTION

Global goat meat production and consumption significantly impact the food industry, driven by its nutritional benefits, such as lower fat content and a favorable amino acid profile. The worldwide production of goat meat has risen from 5.1 million tons in 1970 to 6.39 million tons in 2021 [1], reflecting increasing demand, particularly in regions where it is a dietary staple. There is a notable shift in consumer perception in Thailand, with a growing demand for goat meat due to increased awareness of its nutritional advantages and government promotion of goat production [2]. Understanding consumer attitudes is crucial for the industry’s success, and positive perceptions can drive market growth. This study aims to explore the bioactive properties of goat meat to enhance its market appeal and contribute to industry growth.

The composition of meat differs across various goat primal cuts due to variations in moisture, protein, fat, ash, amino acids, and muscle fiber content [3]. Moreover, meat is widely acknowledged as an exceptional source of protein due to its well-balanced amino acid profile and easy digestibility. Consequently, it’s reasonable to presume that the nutritional makeup of each primal cut contains distinct peptides, potentially leading to differences in their bioactive properties. Within muscle proteins, bioactive peptides are embedded in their sequences. These peptides can be released and exert beneficial biological effects during the process of gastrointestinal (GI) digestion, facilitated by enzymatic hydrolysis [4]. Numerous studies have reported the presence of bioactive peptides derived from various types of meat, including chicken breast, beef, and pork, following GI digestion [5,6]. While research on food digestion in humans is considered the gold standard due to its reliability, it presents significant challenges in terms of cost and ethical considerations. As a result, *in vitro* models have been employed for many years to replicate the process of food digestion. The European Cooperation in Science and Technology (COST) action International Network for Food Digestibility and Health (INFOGEST) has introduced a standardised static *in vitro* digestion procedure [7], which is now recognised as a practical methodology for evaluating the various stages of food digestion. This method, designed for standard laboratory equipment, aims for broad adoption by researchers. It employs a static digestion technique with consistent meal-to-fluid ratios and pH throughout each step. Bioactive peptides derived after GI digestion play a vital role in maintaining physiological health by traversing the intestinal wall to reach target organs in an active state [8,9]. The passage through intestinal epithelial cells involves hydrolysis by brush border proteases, a process capable of inducing structural changes and fragmenting peptide sequences, ultimately influencing their bioactive properties. To assess peptide transport under controlled conditions, researchers commonly utilize *in vitro* assays with colorectal adenocarcinoma (Caco-2) cell monolayers [8]. Oligopeptides employ various transport mechanisms, including passive transport and transcytosis, whereas di- and tri-peptides depend on transporter 1 (PepT1) [9]. Quantifying bioactive peptides transported across Caco-2 cell monolayers from diverse food sources reveals a range of 0.4% to 15% [8,9].

Bioactive peptides are specific protein fragments that extend beyond their nutritional functions, positively affecting bodily processes and potentially impacting human health. Recent research suggests that these peptides may effectively mitigate the risk of obesity and the onset of type-2 diabetes by suppressing the enzyme dipeptidyl peptidase-IV (DPP-IV), which is present in various organs, including the kidney and the gut. DPP-IV is responsible for breaking down incretin hormones like glucagon-like peptide-1 and gastric inhibitory peptide, which play a vital role in regulating glucose levels by enhancing insulin production and reducing glucagon release. Consequently, one potential approach for managing type 2 diabetes involves inhibiting DPP-IV, and diabetes is a prevalent global health concern often associated with hypertension, underscoring the importance of preventive measures and management for both conditions [10].

Another bioactive characteristic worth mentioning is its function as an angiotensin-converting enzyme (ACE) inhibitor. ACE plays a pivotal role in regulating blood pressure by expediting the conversion of angiotensin I to angiotensin II and deactivating bradykinin. In the context of modern medicine, ACE inhibitors have effectively managed ACE activity and treated hypertension. However, it’s important to acknowledge that synthetic DPP-IV and ACE inhibitors have specific adverse effects, prompting researchers to explore natural alternatives; diabetes and hypertension are the two most prevalent risk factors for atherosclerosis and cardiovascular disease [11]. Recently, scientists have extensively studied bioactive peptides derived from food proteins due to their potential to inhibit DPP-IV and ACE, offering antidiabetic and antihypertensive advantages. These peptides could potentially be further developed into dietary products to enhance health [4–6]. Most of the ACE and DPP-IV inhibitory peptides have been sourced from muscle-based foods, employing various commercial proteases like pepsin, papain, alcalase, and neutrase [12]. Interestingly, peptides can be directly obtained from meat following consumption. For instance, research has explored raw beef, pork, and chicken as potential sources of ACE and DPP-IV inhibitory peptides during the process of GI digestion and absorption [6]. Nevertheless, it’s crucial to note that these findings haven’t directly translated into concrete health benefits because consuming raw meat is uncommon due to concerns about food safety and consumer acceptance.

For safe consumption, it is universally accepted that goat meat requires thorough cooking. This process significantly transforms protein structures, making them more accessible to GI enzymes and forming numerous bioactive peptides. A study by Liu et al [13] demonstrated that cooking goat semimembranosus muscle at temperatures ranging from 50°C to 90°C for 30 min resulted in optimal outcomes at an internal temperature of 70°C. This temperature was found to minimize cooking losses and reduce shear force levels. In a separate study, Sangsawad et al [5] found that cooking Korat chicken breast at 70°C for 30 min resulted in the highest level of GI digestion. The peptides released during this process exhibited the most potent inhibition of ACE. Furthermore, Bıyıklı et al [14] reported that cooking turkey cutlets at varying temperatures (65°C to 75°C for 20 to 60 min) effectively inactivated pathogens, including Listeria spp. These results suggest that cooking at 70°C for 30 min may be sufficient to ensure safety from pathogens. Therefore, examining goat meat cooked at 70°C for 30 min provides a realistic reflection of common consumption practices. However, there is still a vast, unexplored area of research concerning the dual functionality of ACE and DPP-IV inhibitory peptides produced during the GI digestion of cooked goat meat.

Over the past few decades, the Paengkoum research team conducted a significant investigation centered on producing Boer goats using animal feed and evaluating meat quality [15–17]. Unfortunately, there has been a noticeable absence of thorough research and assessment when it comes to understanding the nutritional advantages of goat meat for human well-being. Earlier studies have highlighted substantial distinctions in the chemical composition of goat meat, encompassing various cuts such as the neck, shoulder, breast, loin, and leg [3,18]. As a result, it is reasonable to anticipate that differing muscle tissues derived from distinct primal cuts would yield varying levels of ACE and DPP-IV inhibitory effects after undergoing GI digestion and absorption. To the best of our knowledge, no prior research has been published on the nutritional aspects of goat meat despite its demonstrated dual functionality in reducing hypertension and managing diabetes during *in vitro* GI digestion and absorption. Consequently, the main objective of this study is to delve into the nutritional value of goat meat and its potential as a source of bioactive peptides during GI digestion and absorption, all in the pursuit of enhancing human health.

## MATERIALS AND METHODS

### Chemicals

Pepsin, pancreatin, ACE, DPP-IV, gly-pro-p-nitroanilide (GPN) hydrochloride, N-[3-(2-furyl)acryloyl]-phe-gly-gly (FAPGG), trifluoroacetic acid (TFA), acetonitrile (ACN), Lowry reagent, 2,4,6-trinitrobenzene sulfonic acid (TNBS), trypsin (0.25%), and fetal bovine serum (FBS) were all obtained from Sigma-Aldrich Company in St. Louis, MO, USA. Cell culture inserts (24 wells) were also purchased from Corning in Mississauga, ON, Canada. Caco-2 (HTB-37) cells at passage 20 were used for cell culture, and they were acquired from the American Type Culture Collection in Manassas, VA, USA. Streptomycin, penicillin, Eagle’s minimum essential mineral (EMEM) medium, Hank’s Balanced Salt Solution (HBSS), 3-(4,5-dimethylthiazol-2-yl)-2,5-diphenyltetrazolium bromide (MTT), and sodium dodecyl sulfate (SDS) were sourced from Invitrogen Canada in Burlington, ON, Canada. It’s important to note that all other compounds utilised in our research met analytical quality standards.

### Sample preparation

**Figure 1:**
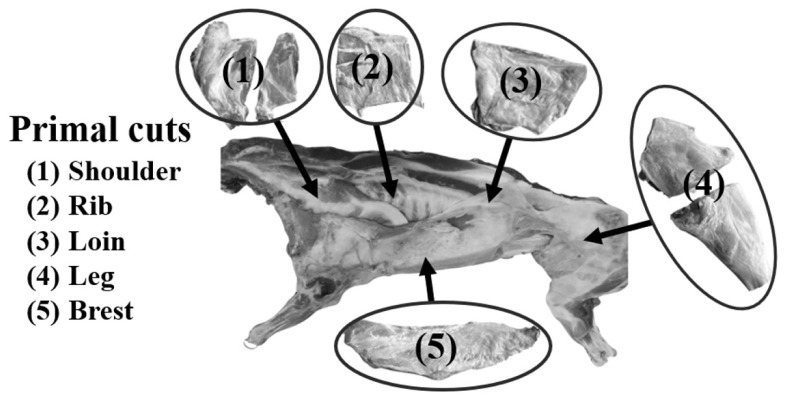
Cutting locations of 5 primal cuts including shoulder, rib, loin, leg, and breast.

The meat samples used in this investigation were purchased from SUT Mart, the University shop at Suranaree University of Technology in Thailand. Ten random samples from each primal cut were picked for the experiment, each weighing 1 kg. These primal cuts originated from 10-month-old male Boer goats with an average carcass weight of 30±2 kg, including the shoulder, breast, rib, loin, and leg (Figure 1). Afterward, the meat samples from every primal cut were gathered and vacuum-sealed within 3 h postmortem. These sealed samples were placed in a freezer at −80°C until analysis.

### Chemical compositions

#### Proximate analysis

The goat meat samples were randomly selected, minced, and then analysed to determine their moisture, fat, protein, and ash contents using the AOAC’s procedures [19]. The protein content was determined using a Kjeldahl analyser and a conversion factor of 6.25.

#### Amino acid analysis

The amino acid content was assessed following the procedure outlined in the study by Hamzeh et al [20]. To summarise, 200 mg of goat meat samples were treated with hydrolysis for 24 h at 110°C in a solution comprising 6 M HCl (5 mL) and 1% phenol. The amino acids were then measured using an amino acid analyser that employed the ninhydrin postcolumn technique (Biochrom 30, Biochrom Ltd., Cambridge, UK). The amino acid content obtained was reported as g/100 g of total amino acids.

#### Gel electrophoresis analysis

Thermal processing is pivotal in food preparation, particularly for dishes centered around muscle-based ingredients. Our research employed a cooking method following Sangsawad et al [5,21], which was used to cook the primal cuts of goat. This cooking procedure entailed cutting the muscle into pieces measuring 3×3×3 cm^3^ each, placing them in nylon vacuum bags, and then subjecting them to a 30-min boil at 70°C. Following cooking, the meat was cooled to 25°C and subsequently frozen, with storage at −20°C for future use. Sodium dodecyl sulfate-polyacrylamide gel electrophoresis (SDS-PAGE) was utilised to scrutinise the protein composition of the goat meat, following the protocol described by Sangsawad et al [21]. For this analysis, 1 g of cooked meat in 5% SDS (19 mL) was homogenised and subjected to a 10-min heat treatment at 95°C and after centrifugation at 10,000 g for 10 min, then combined the supernatant with the sample buffer in a 1:1 ratio. Subsequently, 15 μg of protein was loaded onto 10% acrylamide gels for electrophoresis, maintaining a constant voltage of 120 V for 60 min to facilitate protein separation. The gel was then stained with solution A (0.125% Coomassie Brilliant Blue R-250) for 120 min and subjected to destaining with solution B (25% ethanol and 10% acetic acid) for 60 min, repeating this process twice.

### *In-vitro* gastrointestinal digestion of the cooked meats

The experiment simulated human GI digestion of food proteins *in vitro*, following the INFOGEST protocol [7], to evaluate the digestibility of cooked goat meat. This process involved two distinct phases: first, the stomach phase homogenised 1 g of cooked meat in simulated gastric fluid with a pH of 3 (using 10 mL). Subsequently, a pepsin solution was added with a final concentration of 2,000 U/mL and CaCl_2_ with 0.15 mmol/L. The samples were then incubated at a constant shaking speed of 120 rpm and a temperature of 37°C for 2 h. The pH was adjusted to 7.0 to stop pepsin activity. Second, the small intestine phase: taking 10 mL of simulated intestinal fluid to the material that had undergone digestion in the stomach phase. Following this added pancreatin (with a final concentration of trypsin activity at 100 U/mL) and CaCl_2_ (0.3 mM). The samples were subjected to another round of incubation, with constant shaking at 120 rpm, at a temperature of 37°C for 2 h. The process was terminated by heating the samples for 15 min at 95°C. Then, the supernatant was collected after centrifugation for 10 min at 10,000 g at 4°C. The experiment employed a specific equation that relies on the α-amino content of the sample to determine the digestibility of the cooked meat. This content was determined using the TNBS assay according to Adler-Nissen [22], with leucine serving as the standard:


(1) $$Digestibility\;(\%)=\frac{\alpha A_{4h}-\alpha A_{0h}}{\alpha A_{total}}\times 100$$

Where αA4h and αA0h represent the α-amino content of the digest after 4 h and 0 h of GI digestion, respectively, while αAtotal denotes the total α-amino content of the substrate sample, αAtotal was determined by subjecting the substrate sample to hydrolysis using 6 M HCl at 120°C for 24 h.

### Bioactive properties of peptide derived after gastrointestinal digestion

Our study assessed the bioactivity of peptides derived from cooked goat meat after 4-h digestion in the GI system, with particular attention to their ability to inhibit ACE and DPP-IV. Moreover, the peptides that showcased the greatest activity were utilised as our selection criteria.

The ACE inhibitory activity was assessed according to the procedure outlined by Sangsawad et al [4]. The experiment employed the protocol outlined to assess ACE inhibitory activity. In brief, a 20 μL peptide sample was combined with 10 μL of ACE at 1 mU/mL concentration in a 96-well microplate and then incubated at 37°C for 5 min. Then, 80 μL of 0.5 mM FAPGG substrate was added, and incubated the mixture at 37°C for 30 min. The absorbance at 340 nm was measured using a microplate reader (Varioskan LUX; Thermo Scientific, Vantaa, Finland). As a positive control, DI water was substituted for the sample. The reaction rate (slope) was utilised as an indicator of activity, and the inhibitory activity was calculated using the following formula:


(2) $$ACE\;inhibition\;(\%)=\frac{{Slope}_{positive\;control}-{Slope}_{test\;sample}}{{Slope}_{positive\;control}}\times 100$$

The DPP-IV inhibitory activity was assessed according to the procedure outlined by Sangsawad et al [4]. In short, a 20 μL peptide sample was mixed with 10 μL of DPP-IV (10 mU/mL) in a 96-well microplate and incubated at 37°C for 5 min. Subsequently, 50 μL of the substrate, 30 mM GPN, was added, and the mixture was incubated at 37°C for 30 min. The absorbance was continuously tracked at 405 nm using a microplate reader (Varioskan LUX; Thermo Scientific, Finland). DI water was substituted for the sample to serve as a positive control. The reaction rate (slope) was employed to indicate the activity, and the inhibitory activity was determined using the following formula:


(3) $$DPP\;inhibition\;(\%)=\frac{{Slope}_{positive\;control}-{Slope}_{test\;sample}}{{Slope}_{positive\;control}}\times 100$$

### The molecular weight distribution of peptides derived after gastrointestinal digestion

Size exclusion chromatography was employed to analyse the GI digests (100 μL) with a Superdex Peptide 10/300 GL column to determine their molecular weight. The elution (30 mL) was performed using an AKTA Purifier (GE Healthcare, Piscataway, NJ, USA) by using a mixture of 30% ACN and 0.1% TFA in DI water, operating in an isocratic mode with a 0.7 mL/min flow rate. Then, the peptide profile was identified by measuring UV215 nm absorbance. The study utilized common substances such as cytochrome-c, aprotinin, synthetic peptides, and tyrosine to determine the molecular weight distribution of peptides.

### *In-vitro* bioavailability of the peptide derived after gastrointestinal digestion

Caco-2 cells serve as a widely employed model in the examination of the bioavailability of peptides, as they closely emulate the characteristics of the intestinal epithelium [23]. Caco-2 cells (passages 20 to 30) were seeded into 96-well culture plates at 1×10^5^ cells/cm^2^ density. These cells were cultivated in EMEM media supplemented with 10% FBS and antibiotics (100 μg/mL penicillin and 100 U/mL streptomycin) while being maintained at 37°C in a 5% CO_2_ environment. Over 21 days, the medium (100 μL) was regularly replaced every 2 to 3 days. Our principal objective centred on evaluating the cytotoxicity of peptide samples, specifically GI digests originating from the shoulder and loin. The culture medium (100 μL) was substituted with GI peptides dissolved in EMEM, and the concentrations were adjusted from 0 to 1,000 μg/mL. Following this step, the cells were subjected to a 24-h incubation at 37°C in a 5% CO_2_ atmosphere. Subsequently, after an additional 4-h incubation in the dark, 100 μL of MTT solution (0.05 mg/mL in EMEM) was added. The resultant formazan product was quantified using a microplate reader (Varioskan LUX; Thermo Scientific, Finland) at a wavelength of 570 nm after adding 100 μL of 10% SDS in 0.01 M HCl. The entire mixture underwent an overnight incubation.

The study on peptide transport followed the procedures outlined by Sangsawad et al [24]. The initial step involved seeding Caco-2 cells into 24-well cell culture inserts at 2.5 ×10^5^ cells/cm^2^ concentration in 0.7 mL of EMEM applied to the basal side. As previously specified, the culture medium was meticulously refreshed every 2 to 3 days for 21 days. Following this incubation period, the cells underwent a two-stage treatment: they received two rounds of rinsing with HBSS and were subsequently allowed to equilibrate for 1 h at 37°C. A Millicell ERS-2 volt-ohmmeter (EMD Millipore, Darmstadt, Germany) was employed to assess transepithelial electrical resistance (TEER). Only monolayers exhibiting a TEER value exceeding 250 Ω U/cm^2^ were selected for further investigation in the transport study.

The peptide transport study was initiated by adding 0.3 mL of test samples (500 μg/mL) dissolved in HBSS to the apical side and an additional 0.7 mL of HBSS to the basal side. Subsequently, these plates were placed in an incubator set at 37°C, with an atmosphere containing 5% CO_2_, for 2 h. Following this incubation period, measurements of the peptide content in the apical compartment were conducted both before (0 h) and after (2 h) of incubation. Meanwhile, the peptide content in the basal compartment was quantified after a 2-h incubation. Our estimation of peptide content followed the Lowry method following the protocol outlined by Sangsawad et al [24]. Additionally, for the computation of bioavailability, the protein content present in both the apical and basolateral areas was utilised, employing the subsequent equation:


(4) $$Bioavailability\;(\%)=\frac{Total\;protein\;{content}_{in\;basal\;side\;at\;2-h}}{Total\;protein\;{content}_{in\;apical\;side\;at\;2-h}}\times 100$$

The ACE and DPP-IV inhibitory activities of the transported peptide on the apical and basal side were examined after 2 h of incubation and expressed as the IC_50_ value.

### Statistical analyses

All studies were performed in triplicate, and the data were analysed through a one-way variance analysis. Duncan’s multiple ranges mean comparison test with SPSS 17.0 software (SPSS Inc., Chicago, IL, USA) was utilized to investigate significant differences in mean values within a 95% confidence interval.

## RESULTS AND DISCUSSION

### The chemical composition of goat meat varies across different primal cuts

#### Proximate composition

The analysis of goat meat composition, detailed in Table 1, provides valuable nutritional insights. Notably, the protein content in goat meat emerges as a predominant component, with concentrations of 20.35%, 19.79%, and 19.30% in the shoulder, loin, and rib cuts, respectively. To better understand these findings, it is instructive to compare them with protein contents in other meats reported by Mohammed et al [25], which include camel (21.83%±1.33%), beef (20.64%±1.03%), mutton (21.62%±1.77%), and chicken (22.73%±0.68%). While goat meat protein levels fall within this range, the shoulder, loin, rib, and leg cuts are substantial protein sources. Turning to fat composition, the fat content in goat meat’s shoulder, loin, rib, and leg ranges from 1.44% to 2.29%. Our results align with the research of Shija et al [26] and Sen et al [27], who reported protein and fat levels in goat meat within the approximate range of 15% to 25% and 2% to 8%, respectively. In comparison, Mohammed et al [25] found varying crude fat levels in other meats, such as fresh camel (1.51%), beef (6.83%), mutton (4.56%), and chicken (0.89%). These comparisons highlight the relatively lean nature of goat meat, aligning with Utrera et al [28] recommendation of maintaining crude fat levels below 3% to reduce lipid oxidation during storage. Notably, our study identified the breast sample as an exception, surpassing the recommended fat limit with a content of 4.35. This observation contrasts with findings in other meats, underscoring the importance of cut-specific analysis. Regarding ash composition, goat meat samples consistently contained less than 2%. A parallel can be drawn with Mohammed et al [25] results for camels (0.83%), beef (1.53%), mutton (0.84%), and chicken (0.96%). These similarities affirm our findings’ overall alignment with broader meat composition patterns.

In summary, the comparison with the results of Mohammed et al [25] contextualizes the nutritional values of goat meat within the broader spectrum of meats. It reinforces the distinctive qualities of specific goat cuts. The high protein and comparatively low-fat content characterize the shoulder, loin, rib, and leg cuts, making them promising protein sources with potential benefits during gastrointestinal digestion and absorption.

#### Amino acid composition

The amino acid composition of five meat samples is presented as a percentage (g/100 g of total amino acids) in Table 2. Consistent intake of essential amino acids (EAAs) is vital for sustaining human health, as the body does not naturally synthesize these compounds and must be obtained through dietary sources. EAAs encompass histidine, isoleucine, leucine, lysine, methionine, phenylalanine, threonine, valine, and tryptophan. Across the spectrum of five different meat varieties, EAAs exhibit variability from 36.74% to 42.40%. The loin (42.40%) and leg (42.35%) meats are particularly noteworthy, distinguished by their high concentration of essential amino acids. Leucine, lysine, valine, and isoleucine contribute the highest percentages to this composition. To provide further context, we compare previously published results on the camel, beef, mutton, and chicken amino acids by Mohammed et al [25]. Their findings align closely with our results, reinforcing the significance of loin and leg cuts as substantial sources of EAAs, particularly leucine, lysine, valine, and isoleucine.

Considering insights into non-essential amino acids (NEAA) of Mohammed et al [25], specifically in camel meat, we find that camel meat is rich in NEAA, including glutamic acid, proline, arginine, glycine, and aspartic acid. The substantial NEAA content in camel meat is mirrored in our study, particularly in the breast and leg cuts. These results suggest that goat meat can be a valuable source of NEAA, especially in specific cuts. In summary, the comparison with Mohammed et al’s results [25] provides valuable insights into the amino acid composition of goat meat, reinforcing the significance of essential and non-essential amino acids in different meat sources. The rich concentration of amino acids, particularly in the loin and leg cuts, underscores the nutritional value of goat meat, potentially contributing to dietary essential amino acid intake and customer satisfaction. Further exploration of amino acid profiles in different meats contributes to a broader understanding of their impact on human health and well-being.

When comparing all amino acids in Table 2, the top four were glutamic acid, aspartic acid, leucine, and lysine, respectively. What makes these results intriguing is the revelation of the highest percentage of glutamic amino acids, which falls within the range of 17.3% to 18.3%. Rotola-Pukkila et al [29] reported that the presence of glutamic acid in meat is responsible for imparting a delightful umami flavor, and it’s important to underscore the potential impact of this on customer acceptance after consumption.

Additionally, within the framework of promoting healthy dietary practices, our investigation uncovered that leucine, isoleucine, and lysine emerged as the most prevalent among the three branched-chain amino acids (BCAAs). Notably, BCAA content typically tends to be higher in animal-based proteins than in plant-based sources, with red meat exhibiting the highest concentrations [30]. Furthermore, BCAAs play a pivotal role in enhancing muscle protein synthesis and have been found to activate numerous regulatory mechanisms linked to increased mitochondrial metabolism and improve cellular metabolism [31]. Our findings showed minor disparities in BCAA levels across five different meat types, collectively accounting for 18.75% to 19.7% Notably, these levels were substantially higher than those found in chicken breast at 5.14% [32] and beef at 14.5% [33].

### SDS-PAGE pattern of goat meat in various primal cuts

Thermal processing is crucial in food preparation, particularly regarding muscle tissue dishes. Heat application is a key factor that leads to the denaturation of proteins, affecting the chemical makeup of amino acid residues and the micromorphology and aggregation of proteins. This insight is supported by Liu et al [34] research. Furthermore, it’s worth noting that elevated surface hydrophobicity has been observed to enhance protein degradation by proteases. In addition, under hyperoxidative conditions, proteins may form intermolecular crosslinks and aggregates, potentially reducing their susceptibility to enzymatic proteolysis. This phenomenon is highlighted in the study by Domian and Mańko-Jurkowska [35].

**Figure 2:**
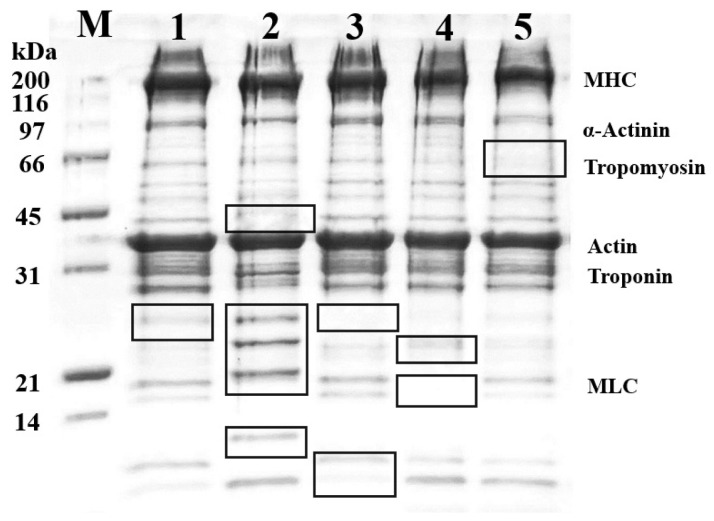
Sodium dodecyl sulfate-polyacrylamide gel electrophoresis (SDS-PAGE) profile of cooked goat meat with various muscle types: (M) protein standard markers, (1) Breast, (2) Shoulder, (3) Loin, (4) Rib, and (5) Leg.

In our research, the meats were cooked at a heating temperature of 70°C for 30 min. The protein pattern of the cooked goat meats was separated using the SDS-PAGE technique and is shown in Figure 2. The protein profiles of all samples were similar, with the main proteins in goat meat being myosin heavy chain and actin, having molecular weights of approximately 220 kDa and 42 kDa, respectively. However, observing the Black square frame, the gel displayed varying amounts and intensities of muscular proteins. The most significant protein pattern was observed with a molecular weight below 31 kDa, particularly the protein band between 21 and 31 kDa in the shoulder, which differed from the others. According to the previous research conducted by Sangsawad et al [21], muscle proteins undergo some modifications, including degradation and structural alterations, under mild thermal treatment. Thus, these results indicate that cooking goat meat at mild thermal temperatures causes the breakdown and modification of proteins in goat muscle.

### The protein digestibility of cooked goat meat in different primal cuts during the process of GI digestion

**Figure 3:**
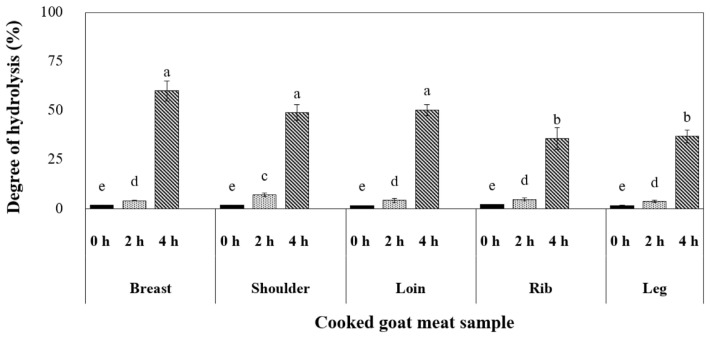
The protein digestibility (degree of hydrolysis) in different primal cuts of cooked goat meat. ^a–e^ Distinct superscript letters mark substantial value differences (p≤0.05).

The experiment was conducted to simulate the digestive processes in the GI tract using cooked goat meat samples. In the initial two h, the digestion process resembled the conditions in the stomach. Our findings from Figure 3 illustrated that pepsin, a gastric enzyme, had a limited effect on breaking down muscle proteins, with the shoulder sample displaying the highest degree of hydrolysis (7.0%). As the process continued for an additional two h in the small intestine, pancreatic enzymes enhanced the extent of digestion, ranging from 40% to 60%. This phase is crucial for muscle protein breakdown, and the breast, shoulder, and loin samples exhibited the most significant hydrolysis levels. According to a previous report by Sangsawad et al [24], gentle heat treatment can improve the digestibility of chicken meat. Our research suggests that cooking breast, shoulder, and loin cuts at 70°C for 30 min may represent the optimal approach, potentially leading to enhanced protein digestion.

### The molecular weight distribution of peptides derived from simulated GI digestion of cooked goat meat

**Figure 4:**
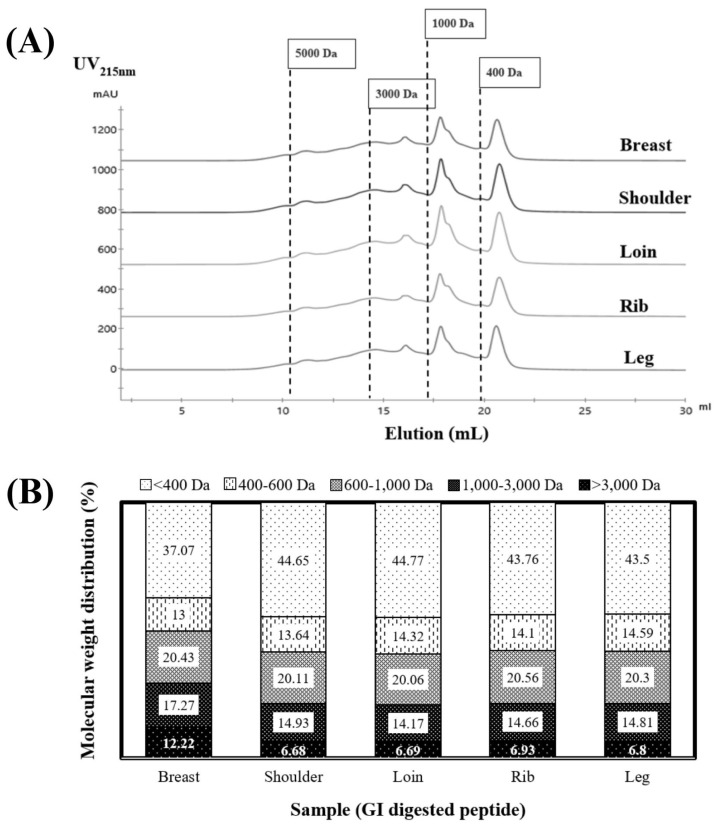
The chromatogram of peptides derived from cooked goat meat after 4 h of gastrointestinal (GI) digestion with separated using size exclusion chromatography (a), the calculated molecular weight distribution (b).

Figure 4 provides a visual representation of the molecular weight distribution of peptides resulting from the 4-h GI digestion of cooked goat meat samples. These peptides’ size distribution ranged from <400 to 5,000 Da, as depicted in Figure 4A. Notably, sharp peaks in elution volume at 18 and 21 mL signified the presence of smaller peptides measuring <1,000 Da. When we examined the proportions of each peptide size in Figure 4B, it became evident that the majority fell within the smallest size, <400 Da, making up 37% to 44% of the total. These results corroborate the research conducted by Martini et al [6], in which they documented that a substantial proportion exceeding 40% of the peptides exhibited molecular weights below 500 Da in the context of GI digestion. An important insight is that peptides <400 Da likely include di- and tri-peptides, which are more stable and readily absorbed by epithelial cell receptors through the human peptide transporter 1 [36].

Moving to the second fraction, it encompassed peptides sized between 400 and 1,000 Da, consisting of tetra- and pentapeptides. These compounds are typically absorbed via the paracellular tight junction [37]. In our results, the peptide profiles of the shoulder and loin demonstrated the highest proportions, accounting for approximately ~44% of the <400 Da-sized peptides and ~34% of the 400 to 1,000 Da-sized peptides (Figure 4B). Conversely, the breast sample exhibited the lowest percentage. This variation did not appear to be linked to the degree of hydrolysis, suggesting potential differences in peptide structure and sequence among the samples. In summary, these findings underscore the significance of cooked shoulder and loin as sources of readily digestible and smaller peptides.

### The bioactivity of peptides derived from cooked goat meat after simulated GI digestion in various primal cuts

ACE inhibitory peptides, derived from food proteins, have demonstrated promise in preventing and controlling hypertension. Another bioactive property, DPP-IV, plays a critical role in glucose metabolism by deactivating incretins. In this specific context, DPP-IV inhibitors extracted from food proteins hold the potential to serve as effective glycemic regulators, possibly preventing the initiation of type 2 diabetes through customized nutritional strategies.

Table 3 presents the outcomes of our research, which focused on the bioactive properties of peptides derived from the GI digestion of cooked goat meat. After a digestion period of 4 h, we observed that all peptides derived from the meat effectively inhibited ACE and DPP-IV enzymes. This significant finding suggests that all primal goat meat cuts could potentially serve as a rich source of bioactive peptides, promoting health benefits. Among the tested samples, peptides derived from the shoulder and loin cuts of the cooked goat meat exhibited the highest ACE and DPP-IV inhibition activity levels. These results indicate their superior potency in comparison to peptides from other cuts. Conversely, peptides from the leg cut displayed the lowest activity levels. We also noted a distinct variation in the bioactivity of peptides derived from different meat cuts. This variation might be attributed to the unique amino acid profiles, peptide chain sizes, and specific peptide sequences released from the parent protein during GI digestion inherent to each cut. These variations underscore the need for further characterization in subsequent studies to understand these peptides’ bioactive potential fully. This research opens up new avenues for developing functional foods leveraging the health-promoting properties of these bioactive peptides. Furthermore, our results indicated a correlation between the ACE and DPP-IV inhibitory activities of the peptides derived from the cooked shoulder and loin and their molecular weight distribution. These peptides were found to contain the highest percentage of short-chain peptides, as depicted in Figure 4B (with a molecular weight of <400 Da). Previous research by Ketnawa et al [38] commonly observed that lower-molecular-weight peptides were linked to the inhibition of ACE and DPP-IV activities.

In comparison to prior studies on bioactive peptides derived from GI digestion, the peptides extracted from cooked shoulder and loin demonstrated superior ACE inhibitory activity at the same concentration compared to sericin protein, cooked chicken breast, pork, and beef, with values of 20%, 55%, 40%, and 30%, respectively [4,5,39]. Additionally, the peptides derived from cooked shoulder and loin exhibited higher DPP-IV inhibitory activity than hemoglobin, fish gelatin, and pea protein, with values of 5%, 10%, and 30%, respectively [40].

Our findings reveal that after GI digestion, the bioactive peptides derived from the cooked shoulder and loin exhibited the highest degree of bioactivity, substantiated by their capacity to inhibit ACE and DPP-IV. As a result, based on these outcomes, the cooked shoulder and loin are identified as the ideal primal cuts for providing bioactive peptides capable of eliciting antihypertensive and antidiabetic effects upon ingestion.

### Bioavailability of the peptides derived after simulated GI digestion of the cooked shoulder and loin

#### Cytotoxicity of the peptides

**Figure 5:**
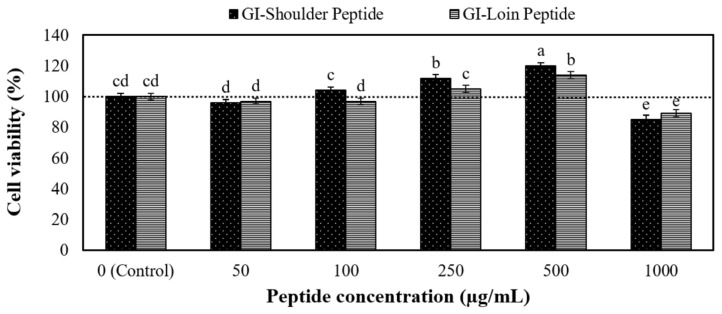
Cell viability of Caco-2 cells was assessed by exposure to peptide concentrations ranging from 0 to 1,000 μg/mL of the cooked shoulder and loin. ^a–e^ Means with different letters are significantly different (p≤0.05).

The peptides obtained from the GI digestion of cooked shoulder and loin underwent desalting before subjecting them to cytotoxicity and *in vitro* bioavailability testing. Figure 5 illustrates the cytotoxicity of these peptides towards Caco-2 cells. Notably, concentrations ranging from 0 to 500 μg/mL exhibited no cytotoxic effects on the Caco-2 cells. However, a mitogenic effect became evident at 250 and 500 μg/mL peptide concentrations. In addition, the relative TEER values showed that monolayer integrity was maintained, even at concentrations as high as 500 μg/mL. These outcomes suggest that the hydrolysate 0–500 μg/mL is likely biocompatible and poses no harm to epithelial cell lines. These findings align with the results of the whey protein peptide test [41] and the chicken muscle protein test Sangsawad et al [21]. As a result, we have concluded that a peptide concentration of 500 μg/mL represents the maximum concentration suitable for the next bioavailability test.

#### Bioavailability and bioactive properties of the peptides

The peptides obtained through GI digestion were applied to the apical surface of the Caco-2 cell monolayers at a concentration of 500 μg/mL for 2 h. Afterwards, the transported peptide samples were collected and subjected to analysis. Peptide concentrations were measured on the cell surfaces’ apical and basal sides. This assessment aimed to determine the extent of peptide uptake compared to the initial concentration. Our study’s findings emphasize that peptides resulting from the GI digestion of cooked shoulder and loin meats exhibit bioavailability. Specifically, the GI peptide sample from the cooked lion exhibited a bioavailability of 12.45%. This percentage was notably higher than the peptide obtained from the cooked shoulder, which showed a bioavailability of 10.32%, as indicated in Table 4. The heightened permeability may be attributed to smaller peptides formed in the cooked loin through sequential digestion by pepsin and pancreatin (Figure 4b). However, the results of this experiment indicate that both peptides had very low permeability. According to previous studies on peptide permeability, casein peptides with molecular weights ranging from 0.5 to 1.6 kDa typically exhibit a permeability range of 9.54% to 10.66%, whey peptides have been found to have a permeability range of 0.2% to 2.5% [42]. It’s worth noting that Caco-2 cell monolayers are recognized as being more impermeable than actual human intestinal cells. Consequently, we can anticipate that the absorption of these peptides *in vivo* will likely be higher compared to what we observed in the Caco-2 cell monolayers.

The bioactivity of peptides was evaluated before peptide transport (apical-0 h, representing the peptide after GI digestion) and after the peptides had undergone transport (basal-2 h, representing the peptides had permeated the intestinal barrier). We assessed their bioactivity by examining their ability to inhibit ACE and DPP-IV. The results of our experiment regarding the transportation or absorption of peptides in the GI tract showed a notable reduction in the ACE and DPP-IV inhibitory activity of both peptides. This reduction led to an increase in the IC_50_ value, as outlined in Table 4. According to the previous research of Fleury et al [40], the IC_50_ values of GI peptides derived from fish gelatin, hemoglobin, casein, and pea protein increased after passing through the Caco-2 cell experiment. It’s worth noting that during the transport of these peptides across the intestinal epithelial cells, brush border proteases can further modify the oligopeptides generated from GI digestion. In line with previous findings, specific peptides, such as KPLLCS, KPLL, ELFTT [43], and IPI [42], have been shown to undergo cleavage, leading to changes in their peptide structure and subsequently impacting their biological activity. When comparing the bioactivities of these permeable peptides, a noteworthy observation emerges: the permeable peptide derived from cooked loin exhibits a lower IC_50_ value of ACE and DPP-IV inhibitions than its counterpart from the cooked shoulder. Thus, this disparity suggests that the cooked loin may possess a greater potential for biological activity.

**Figure 6:**
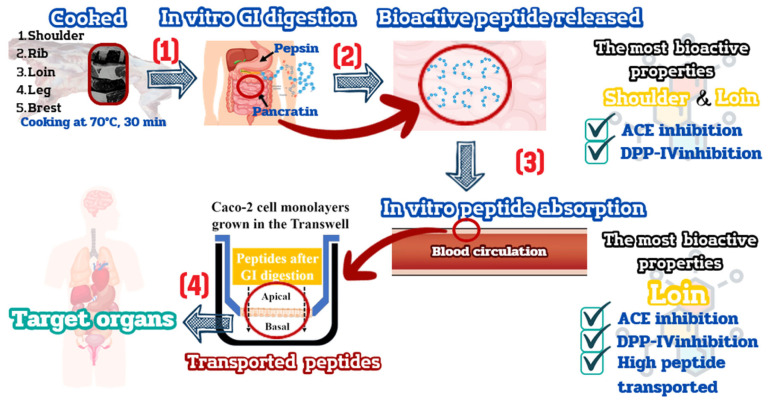
Schematic representation of bioactive peptides derived after gastrointestinal (GI) digestion and absorption through Caco-2 cell monolayers from cooked goat meat.

To comprehensively interpret the outcomes at each stage, Figure 6 provides a schematic representation of bioactive peptides exhibiting significant ACE and DPP-IV inhibition during the gastrointestinal digestion and absorption of cooked goat primal cuts. Our investigation underscores that the variability observed in goat primal cuts stems from differing rates of peptide permeation. Specifically, consuming cooked loin results in the highest peptide permeability, showcasing superior bioactive activity through ACE and DPP-IV inhibition. Substantial evidence supports the observation that peptides when transported *in vitro* through Caco-2 cell monolayers, manifest *in vivo* ACE and DPP-IV inhibitory activity. Notable examples include AAATP from dry-cured ham [44] and LVYPFTGPIPN, HLPLP, IAK, YAKPVA, and WQVLPNAVPAK derived from milk casein peptides [45], demonstrating ACE inhibition properties. Furthermore, studies by Garzón et al [46] and Pei et al [47] illustrate that brewer spent grain peptides and VPLVM peptides from broccoli exhibit DPP-IV inhibitory effects. Consequently, it is reasonable to infer that the GI peptide derived from cooked loin and its breakdown fragments might potentially reach target tissues and exert functional effects *in vivo*.

## CONCLUSION

The study shows that loin is highly nutritious due to its high protein content, essential amino acids, and low-fat levels. Moreover, all the cooked primal cut samples displayed a significant protein structure, as evidenced by the SDS-PAGE results. During GI digestion, all samples were cooked and digested, resulting in most peptides having a small molecular size of <400 Da, which might render them easily absorbable. Notably, peptides derived from the cooked shoulder and loin muscles accounted for over 40% of these peptides and exhibited the most potent ACE and DPP-IV inhibitions. Following the simulation of peptide absorption using cultured intestinal cells (Caco-2 cell monolayers), it was discovered that the GI peptide from the cooked loin exhibited higher levels of bioavailability and dual-bioactivity of ACE and DPP-IV inhibition. Consequently, this study affirms that cooked goat loin (70°C for 30) min, emerges as a source of bioactive peptides with ACE and DPP-IV inhibitory activities; these two bioactive properties correlated with preventing type 2 diabetes and hypertension. Nonetheless, further research is imperative to identify the specific amino acid sequences responsible for these observed activities and to conduct animal and human feeding experiments to explore the therapeutic effectiveness of peptides derived from the cooked loin.

## Figures and Tables

**Table 1:** Proximate composition of goat meat (g/100 g of raw meat)

Sample	Moisture	Protein	Fat	Ash
Breast	77.76^ab^	16.26^c^	4.35^a^	1.09^c^
Shoulder	77.45^ab^	20.35^a^	2.29^b^	1.57^a^
Loin	76.46^b^	19.79^ab^	1.74^d^	1.43^ab^
Rib	78.38^a^	19.30^ab^	1.90^c^	1.28^b^
Leg	78.39^a^	18.26^b^	1.44^e^	1.41^b^

The values were calculated using the mean and standard deviation from three replicates.

a–e Means in the column with different letters differ at p≤0.05.

**Table 2:** Amino acid composition of goat meat (g/100 g of total amino acid)

Amino acid	Breast	Shoulder	Loin	Rib	Leg
Essential amino acid					
Histidine	3.17^c^	2.81^d^	3.86^a^	2.94^d^	3.60^b^
Isoleucine	5.10^b^	5.00^c^	5.25^a^	5.20^a^	5.03^c^
Leucine	8.91^a^	8.80^b^	8.66^c^	8.92^a^	8.39^d^
Lysine	7.09^d^	7.45^c^	7.72^b^	7.97^a^	7.35^c^
Methionine	2.40^c^	2.89^a^	2.44^c^	2.76^b^	2.81^ab^
Phenylalanine	4.14^d^	4.62^b^	4.07^e^	4.23^c^	5.05^a^
Tryptophan	0.28^a^	0.24^b^	0.27^a^	0.28^a^	0.22^b^
Threonine	4.30^b^	3.92^c^	4.63^a^	4.44^b^	4.57^a^
Valine	5.69^a^	5.75^a^	5.50^b^	5.46^b^	5.33^c^
Sum	41.08^c^	41.48^b^	42.40^a^	36.74^d^	42.35^a^
Non-essential amino acid					
Aspartic acid	9.87^b^	9.48^d^	9.65^c^	9.82^b^	10.24^a^
Serine	3.88^b^	3.94^a^	3.78^c^	3.85^b^	3.99^a^
Glutamic acid	17.45^d^	18.06^b^	18.30^a^	17.94^c^	17.30^e^
Glycine	4.95^d^	5.60^a^	5.47^b^	5.36^c^	5.39^c^
Alanine	6.23^b^	6.47^a^	6.13^c^	6.49^a^	6.05^d^
Cystine	0.59^a^	0.64^a^	0.57^a^	0.59^a^	0.45^b^
Tyrosine	3.73^a^	3.42^b^	3.35^c^	3.52^b^	3.57^b^
Arginine	6.60^c^	7.02^a^	6.91^b^	6.10^d^	6.77^c^
Proline	5.92^a^	4.12^c^	3.70^d^	4.41^b^	4.13^c^
Sum	59.22^a^	58.75^b^	57.86^d^	58.08^c^	57.89^d^

The values were calculated using the mean and standard deviation from three replicates.

a–e Means in the row with different letters differ at p≤0.05.

**Table 3:** Bioactivities based on ACE and DPP-IV inhibition of GI-peptides (0.25 mg/mL of the reaction mixture) after 4 h of digestion

Peptide sample^1)^	ACE inhibition (%)	DPP-IV inhibition (%)
Control (GI solution)	26.29^c^	12.75^e^
GIP-Breast	53.14^b^	57.43^b^
GIP-Shoulder	59.63^a^	59.10^a^
GIP-Loin	58.70^a^	58.15^a^
GIP-Rib	54.99^b^	50.42^c^
GIP-Leg	53.55^b^	48.40^d^

ACE, angiotensin-converting enzyme; DPP-IV, dipeptidyl peptidase-IV; GI, gastrointestinal.

1) GIP was the peptide derived after 4-h of GI digestion.

a–e Means in the column with different letters differ at p≤0.05.

**Table 4:** The bioavailability and bioactivity of peptides derived after GI digestion are assessed before (referred to as ‘apical at 0-h’) and after (referred to as ‘basal at 2-h’) their passage through Caco-2 cell monolayers

Condition		Peptides from GI digestion	

		Shoulder	Loin
Bioavailability of peptide (%)		10.32^b^	12.45^a^
Bioactivity of peptide (IC_50_ value, mg/mL in the reaction mixture)			
ACE inhibition	Apical at 0-h	5.21^a^	3.23^b^
	Basal at 2-h	7.52^a^	6.11^b^
DPP-IV inhibition	Apical at 0-h	7.23^a^	4.12^b^
	Basal at 2-h	10.74^a^	7.25^b^

GI, gastrointestinal; ACE, angiotensin-converting enzyme; DPP-IV, dipeptidyl peptidase-IV.

a,b Means in the row with different letters differ at p≤0.05.
